# Myogenic Differentiation from *MYOGENIN*-Mutated Human iPS Cells by CRISPR/Cas9

**DOI:** 10.1155/2017/9210494

**Published:** 2017-04-04

**Authors:** Koki Higashioka, Noriko Koizumi, Hidetoshi Sakurai, Chie Sotozono, Takahiko Sato

**Affiliations:** ^1^Department of Ophthalmology, Kyoto Prefectural University of Medicine, Kyoto, Japan; ^2^Department of Biomedical Engineering, Faculty of Life and Medical Sciences, Doshisha University, Kyotanabe, Japan; ^3^Department of Clinical Application, Center for iPS Cell Research and Application, Kyoto University, Kyoto, Japan

## Abstract

It is well known that myogenic regulatory factors encoded by the *Myod1* family of genes have pivotal roles in myogenesis, with partially overlapping functions, as demonstrated for the mouse embryo. *Myogenin*-mutant mice, however, exhibit severe myogenic defects without compensation by other myogenic factors. MYOGENIN might be expected to have an analogous function in human myogenic cells. To verify this hypothesis, we generated *MYOGENIN*-mutated human iPS cells by using CRISPR/Cas9 genome-editing technology. Our results suggest that MYOD1-independent or MYOD1-dependent mechanisms can compensate for the loss of MYOGENIN and that these mechanisms are likely to be crucial for regulating skeletal muscle differentiation and formation.

## 1. Introduction

In vertebrate embryos, skeletal muscles of the trunk and limbs are derived from the somites, from the dermomyotome which gives rise to myogenic progenitor cells that are directed into the skeletal muscle programme by four myogenic bHLH transcription factors, Myf5, Myod1, Mrf4, and Myogenin (Myog) [1–3]. The myogenic differentiation process in vertebrate embryos is regulated by these factors leading to the formation of multinucleated myotubes and subsequently to the regeneration of skeletal muscle by a reserve of myogenic stem cells in adulthood [4, 5].

Single or compound knockout mice for the genes encoding these myogenic factors have been created to identify their function in myogenesis [3]. Single *Myf5*- or *Myod1*-deficient mice revealed no striking skeletal muscle phenotype [6–8], pointing to overlapping functions between these myogenic determination factors [9–11]. *Mrf4*, which is coexpressed with *Myf5* at the onset of myogenesis, also acts as an early myogenic determination factor [11]. Double and triple mutants for these genes demonstrate their role in determining muscle cell fate. The fourth member of this gene family, *Myog*, is expressed at the onset of muscle cell differentiation. Single *Myog*-deficient mice exhibit severe defects of skeletal muscle formation during development, at a stage when Myod1 and, in many muscles, Mrf4 are also present. This therefore demonstrates that Myog is required for embryonic muscle differentiation, and no redundant or compensatory mechanisms replace its function, unlike for the other myogenic regulatory factors [10, 12–15].

In this study, we have tackled the question of whether these myogenic factors have analogous interrelationships in human myogenesis and, in particular, whether human MYOGENIN (MYOG) is also essential for muscle cell differentiation and muscle fiber formation in myogenic cells derived from human induced pluripotent stem (hiPS) cells. To perform functional experiments, we have used versatile genome-editing technology, with the CRISPR/Cas9 system [16, 17].

## 2. Materials and Methods

### 2.1. Gene Targeting with Human iPS Cells

The *hMYOG*-targeting plasmid vector, pX458-*hMYOG*+189, was constructed using the pX458 vector (Addgene #48138, Cambridge, USA) [17] with ligating oligos (Table 1) as described, and plasmid DNA was introduced into HEK293- or Hu5/KD3-immortalized human myogenic cells [18], with ViaFect reagent (Promega, Madison, USA). The electroporator NEPA21 (NEPA GENE, Chiba, Japan) was used for introducing plasmids into hiPS cells [19].

### 2.2. Cell Culture and Myogenic Differentiation

The hiPS cells were maintained on SNL feeder cells, treated with 10 *μ*g/ml of mitomycin (Sigma, St. Louis, USA) in DMEM (Wako, Osaka, Japan) supplemented with 10% of fetal bovine serum (GIBCO, Grand Island, USA), or expanded in Primate ES cell medium (ReproCELL, Kanagawa, Japan) supplemented with 10 ng/ml of recombinant human FGF2 (bFGF; Wako, Osaka, Japan) and 100 *μ*g/ml of G418 (Nacalai Tesque, Kyoto, Japan). *MYOG*-deficient iPS cells were maintained on SNL feeder cells or iMatrix-511 (Nippi, Tokyo, Japan)-coated plates with StemFit AK03N (Ajinomoto, Tokyo, Japan) under a feeder-free culture system [20].

For the derivation of myogenic cells from hiPS cells, the detailed protocol of Tanaka et al., based on MYOD1 induction [21], was followed. In brief, single iPS cells carrying an inducible MYOD1 activation system were expanded in Primate ES cell medium without bFGF and with 10 *μ*M of Y-27632 (Nacalai Tesque, Kyoto, Japan) for 24 hours and then induced into myogenic cells by adding 500 ng/ml of doxycycline (Dox; Tocris, Bristol, UK). After 24 hours, culture medium was changed into myogenic differentiation medium composed of alpha-MEM (Nacalai Tesque, Kyoto, Japan) with 5% of KSR (GIBCO, Grand Island, USA) and 500 ng/ml of Dox. After 6 days, culture medium was changed into muscle maturation medium, DMEM/F12 (Nacalai Tesque, Kyoto, Japan), with 5% of horse serum (Sigma, St. Louis, USA), 10 ng/ml of recombinant human insulin-like growth factor 1 (IGF-1; PeproTech, Hartford County, USA), and 200 *μ*M of 2-mercaptoethanol (2-ME; Sigma, St. Louis, USA).

To obtain myogenic cells derived from embryonic mesodermal cells, single iPS cells were expanded in StemFit AK03N supplemented with 10 *μ*M of Y-27632. After 2 days, the culture medium was changed into modified mesodermal differentiation medium as described by Loh et al. [22]. Cultured cells were passaged 12 days later and cultured in mesoderm differentiation medium with 10 *μ*M of Y-27632 for 2 days. To initiate myogenic differentiation, medium SF-O3 (EIDIA, Tokyo, Japan), supplemented with 10 ng/ml of bFGF, 10 ng/ml of IGF-1, 10 ng/ml of HGF (PeproTech, Hartford, USA), and 200 *μ*M of 2-ME, was used and changed into myogenic differentiation medium with IGF-1 after 4 days and then with IGF-1 and HGF after 3 days [23]. To obtain more mature myogenic differentiation, culture medium was changed into DMEM/F12 supplemented with 2% of horse serum, 10 ng/ml of IGF-1, and 200 *μ*M of 2-ME 2 weeks later and induced cells were harvested at day 60 [23].

### 2.3. Cell Sorting

Cultured cells transfected with pX458-*hMYOG*+189 were dissociated with TrypLE select (GIBCO, Grand Island, USA) at 37°C for 5 min for detecting transfected cells. Dissociated cells were resuspended with 1% bovine serum albumin in PBS. Cell debris were eliminated with a cell strainer (35 *μ*m; BD, New Jersey, USA), and suspensions were stained with propidium iodide (Molecular Probes, Eugene, USA) to exclude dead cells. Cells were analyzed and collected by a cell sorter using FACSJazz (BD, New Jersey, USA).

### 2.4. Quantitative PCR Analyses

Total RNAs from sorted or cultured cells were extracted using the RNeasy micro kit (QIAGEN, Hilden, Germany). For quantitative PCR analyses, single strand cDNA was prepared using a SuperScript VILO kit (Invitrogen, Carlsbad, USA) as in the manufacturer's protocol. All RT-qPCR reactions were carried out in triplicate using THUNDERBIRD SYBR qPCR Mix (TOYOBO, Osaka, Japan), normalized to the mRNA expression level of ribosomal protein L13A (RPL13A). Primer sequences (5′ to 3′) are listed in Table 2.

### 2.5. Immunofluorescence Assay

Cultured cells were fixed in 4% paraformaldehyde for 10 min at 4°C, permeabilized with 0.2% Triton and 50 mM NH_4_Cl. Fixed samples were pretreated with Blocking One (Nacalai Tesque, Kyoto, Japan) for 30 min at RT and incubated with anti-MYOGENIN (diluted 1 : 100, Santa Cruz Biotechnology, California, USA), anti-MYOSIN HEAVY CHAIN (MYHC, diluted 1 : 200, Santa Cruz Biotechnology, California, USA), anti-TRA-1-81 (diluted 1 : 200, Cell Signaling Technology, Massachusetts, USA), anti-SSEA4 (diluted 1 : 200, Cell Signaling Technology, Massachusetts, USA), anti-OCT4A (diluted 1 : 200, Cell Signaling Technology, Massachusetts, USA), and anti-NANOG (diluted 1 : 200, Cell Signaling Technology, Massachusetts, USA) antibodies in 5% of Blocking One in PBS with 0.1% Tween20 (PBST) overnight at 4°C. After three washes with PBST, cells were incubated with Alexa488-, Alexa594-, or Alexa647-conjugated secondary antibodies (diluted 1 : 500, Molecular Probes, Eugene, USA). Cells were washed with PBST three times and mounted in SlowFade Diamond antifade mountant with DAPI (Molecular Probes, Eugene, USA). Fluorescent images were collected on the software of BZ-X700 (Keyence, Osaka, Japan). Cultured cells were analyzed from triplicate experiments.

## 3. Results

### 3.1. Human MYOGENIN Genomic DNA Editing with the CRISPR/Cas9 System

**Figure 1:**
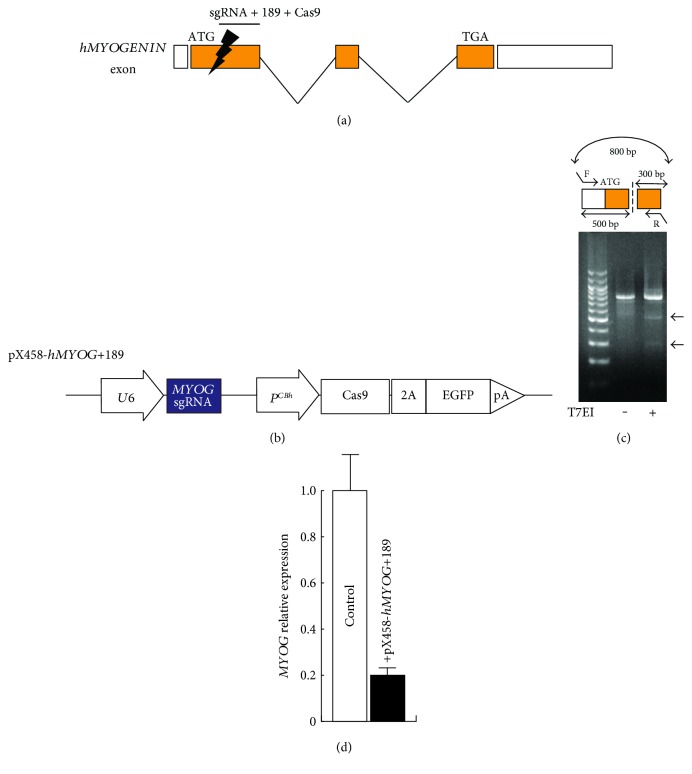
Effect of single guide sequence for *hMYOGENIN* by the CRISPR/Cas9 system. A schematic representation of *MYOG* exons and introns. A candidate position for Cas9 targeting of *MYOG* exon1 (a). pX458-*hMYOG*+189, a construct for driving single guide RNA bound to *MYOG* exon1 and bicistronic expression of both Cas9 and GFP (b). T7 endonuclease I assay for Cas9-mediated cleavage (arrows, 500 bp and 300 bp) on an agarose gel, showing comparable modification of the targeted human *MYOG* genomic fragment in HEK293T cells (c). Relative expression of *MYOG* in Hu5-immortalized human myoblast cells transfected with or without the pX458-*hMYOG*+189 vector. All error bars indicate ±SEM (*n* = 3).

To generate *MYOG*-mutated hiPS cells by double-strand break in *MYOG* exon1 which includes coding sequence (Figure 1(a)), we selected several sequences bound to single guide RNA for targeting by nuclease Cas9 from the CRISPRdirect website as candidates (http://crispr.dbcls.jp, Table 1) [24] and ligated them into the pX458 vector to create the pX458-*hMYOG*+189-editing vector, which targets a unique 20 bp sequence in *hMYOG* exon1 (position 170–192; accaccaggctacgagcgga, Figure 1(b)). The effect of a double-strand break in *hMYOG* genomic sequences was evaluated by heteroduplex PCR fragments, involving the sequences targeted by the pX458-*hMYOG*+189-editing vector, monitored in HEK293 cells and T7 endonuclease I (T7EI). Enzymatic digested PCR bands of 500 bp and 300 bp were observed in T7EI-treated genomic DNA (Figure 1(c)). The data suggested that nuclease Cas9 and single guide RNA target *hMYOG* genomic sequences of exon1. The expression of *MYOG* is initiated in differentiating myogenic cells. To check the amount of *MYOG* transcripts produced from this Cas9 construct, immortalized Hu5/KD3, human myoblasts, transfected with or without the pX458-*hMYOG*+189 vector were differentiated in medium with 2% horse serum for 48 hours. The transcriptional level of *MYOG* was attenuated in differentiated Hu5/KD3 cells (Figure 1(d)). This CRISPR/Cas9 construct for *hMYOG* sequences may not only be effective because of its genomic double-strand break which knocks out *MYOG* expression but may also affect the remaining *MYOG* transcription level.

### 3.2. Generation of *MYOGENIN*-Mutated hiPS Cells

**Figure 2:**
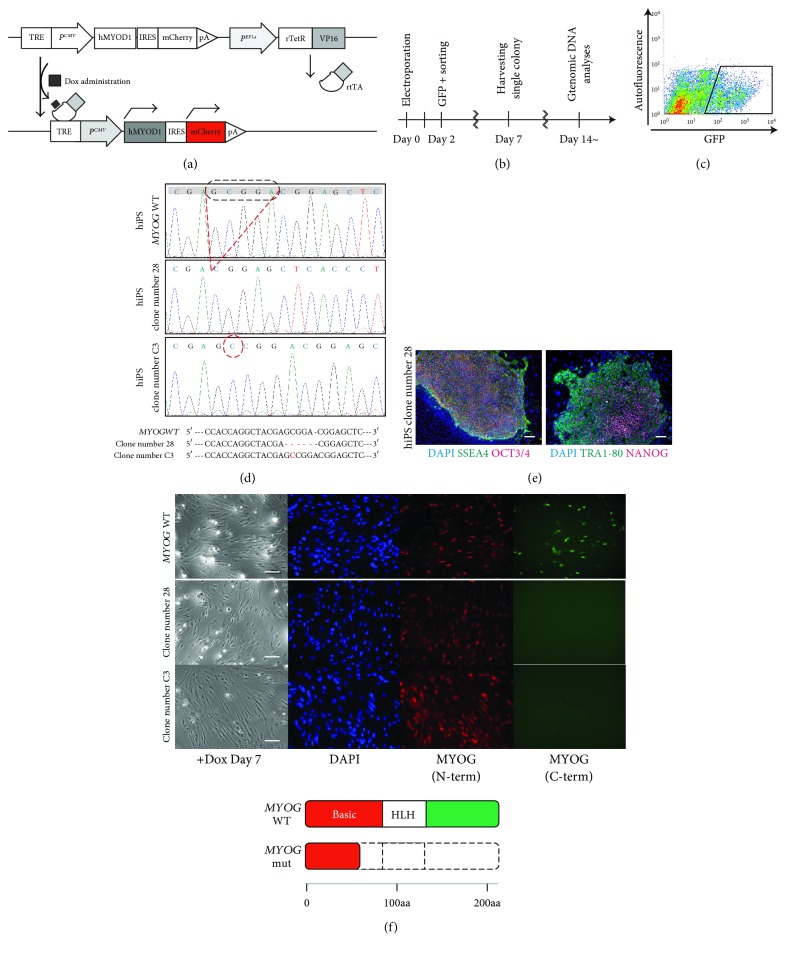
Generation of *hMYOGENIN*-mutated hiPS cells. A schematic model for the induction of myogenic cells derived from hiPS cells by overexpression of *MYOD1* marked with mCherry (red) after administrating Dox (a). A flowchart of the time course for the identification of *MYOG*-mutated hiPS cells (b). FACS analyses to isolate hiPS cells after the introduction of the pX458-*hMYOG*+189 vector (c). Genomic sequence data around the region targeted by pX458-*hMYOG*+189. 5 bp of deletion in clone number 28 and 1 bp of insertion in clone number C3 (dashed lines, (d)). Established hiPS cells were immunostained with undifferentiated pluripotent cell markers, anti-SSEA4 (green in left panel), anti-OCT3/4 (red in left panel), anti-TRA1-80 (green in right panel), and anti-NANOG (red in right panel) antibodies. Nuclei were stained with 4′6-diamidino-2-phenylindole (DAPI, blue). Scale bar, 100 *μ*m (e). Differentiated myogenic cells after Dox treatment for 7 days were immunostained with anti-MYOG N-terminus (N-term, red) and C-terminus (C-term, green) antibodies. Nuclei were stained with 4′6-diamidino-2-phenylindole (DAPI, blue). Scale bar, 100 *μ*m (upper in (f)). Putative MYOG protein structures both in wild-type (*MYOG* WT) and mutated cells (*MYOG* mut) (lower in (f)).

In order to generate *MYOG*-mutated hiPS cells, we used hiPS cells carrying a *MYOD1* expression construct which is inducible with Dox to activate the myogenic programme (Figure 2(a)) [21]. The iPS cells were expanded on SNL feeder-coated plates after electroporation with pX458-*hMYOG*+189 vector for 48 hours, and GFP-positive cells were collected by cell sorting (Figures 2(b) and 2(c)). These cells were plated out to form colonies which were individually picked up. Each clone was screened for further analyses.

We were able to identify 25 clones, which were lacking the wild-type *MYOG* sequences (wild type: 19.4%, heterozygotes; 64.5%, homozygotes; and 16.1%, total screened clones *n* = 31) by checking genomic sequences around the targeted *MYOG* region. Selected clone number 28 or clone number C3 was confirmed to have biallelic on-target frameshift mutations, 5 bp of deletion, and an extra 1 bp of integration in the *hMYOG*-sgRNA and Cas9-targeted region as shown in Figure 2(d). These data suggest that this targeting CRISPR/Cas9 system is sufficiently efficient to knockout both alleles of *MYOG* directly by introducing out-of-frame mutations (lower images in Figure 2(f)). *MYOG*-mutated hiPS cells (clone number 28) were immunostained with undifferentiated pluripotent markers, anti-SSEA4, anti-OCT3/4, anti-TRA1-80, and anti-NANOG antibodies, to evaluate the undifferentiated pluripotent state, and these markers were detected positively in *MYOG*-mutated hiPS cells (Figure 2(e)). To confirm the translation of truncated MYOG protein from these mutated sequences, myogenic cells differentiated from Dox-treated hiPS cells for 7 days were immunoreacted with antibodies against human MYOG N-terminus and C-terminus relatively because *MYOG* mRNAs are transcribed with the extra stop codon, which results from the *MYOG* gene targeting. Myogenic cells derived from wild-type hiPS cells were detected by both of these MYOG antibodies; however, the C-terminus of MYOG was not detected in *MYOG*-mutated hiPS cells (Figure 2(f)).

### 3.3. Skeletal Myogenic Differentiation by MYOD1 Induction

**Figure 3:**
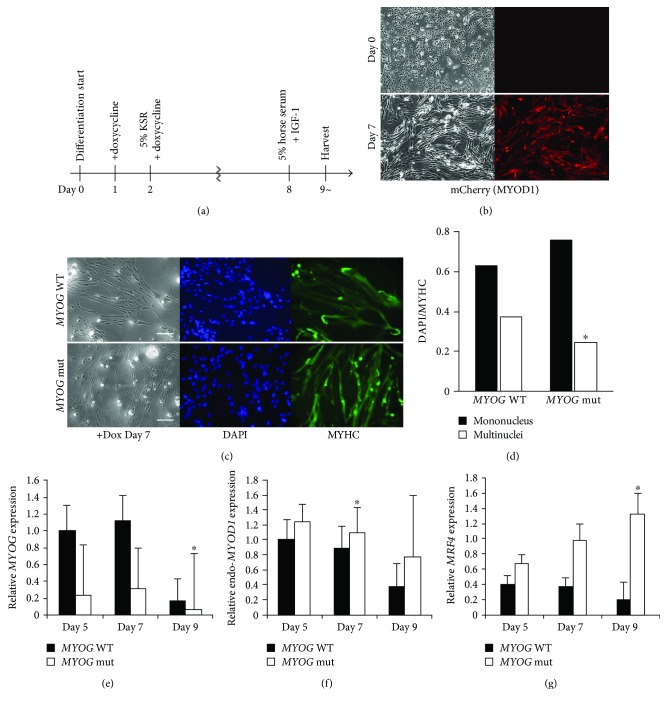
Myogenic differentiation in skeletal muscle cells derived from *MYOGENIN*-deficient hiPS clone number 28 cells. Myogenic differentiation flowchart of the time course (a). Morphological changes and mCherry fluorescent expression after treatment with Dox (b). Differentiated myogenic cells derived from hiPS cells with or without MYOG by Dox treatment for 7 days were immunostained with anti-MYOSIN HEAVY CHAIN (MYHC, green) antibody. Nuclei were stained with 4′6-diamidino-2-phenylindole (DAPI, blue). Scale bar, 100 *μ*m (c). The ratio of DAPI-positive mono or multiple nuclei staining present in single MYHC-positive myofibers derived from wild-type or *MYOG*-mutated hiPS cells (d). Relative expression of transcripts for myogenic regulatory factors, *MYOG* (e), endogenous *MYOD1* (f), and *MRF4* (g), in differentiated myogenic cells treated with Dox for 5, 7, and 9 days. All error bars indicate ±SEM (*n* = 3). *P* values are determined by a *t*-test from a two-tailed distribution. ^∗^*P* < 0.05.

To investigate human MYOG function during myogenic differentiation, MYOD1 was overexpressed in hiPS cells by administrating Dox as shown in Figure 3(a). *MYOD1* expression mimics bicistronic mCherry fluorescence after Dox treatment (Figure 3(b)). Induced myogenic cells derived from hiPS cells were cultured in vitro under differentiation conditions and immunostained for MYHC expression as an indicator of their ability to differentiate into skeletal muscle fibers (Figure 3(c)). Although the rate of myoblast fusion in *MYOG*-mutated hiPS cell clone number 28 was slightly less than that of wild type (Figure 3(d)), terminal differentiation is similar.

To further characterize the differentiation of these myogenic cells, RNA expression of myogenic factors was analyzed by quantitative RT-PCR. The transcript for *MYOG* was downregulated as shown in Figure 1(d) with unknown mechanisms; however, other myogenic factors, notably transcripts of *MYOD*1 or *MRF4*, were upregulated under conditions where *MYOG* is mutated in human myogenic cells (Figures 3(e)–3(g)).

### 3.4. Skeletal Muscle Differentiation via Mesodermal Differentiation In Vitro

**Figure 4:**
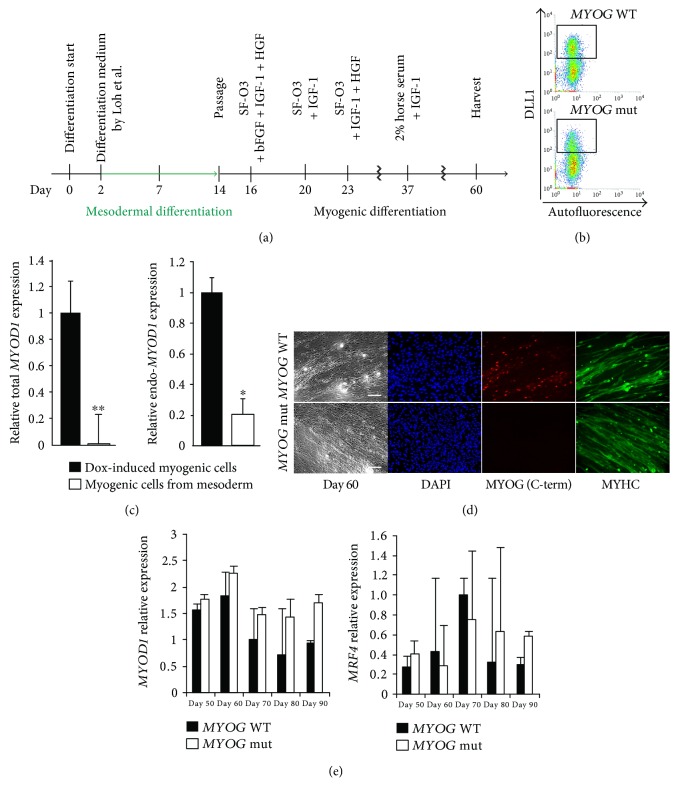
Myogenic differentiation from mesodermal precursors derived from *MYOGENIN*-deficient hiPS clone number 28 cells. Mesodermal and myogenic differentiation flowchart of the time course for muscle cells derived from hiPS cells (a). FACS analyses of DLL1-positive mesodermal cells derived from hiPS cells (b). Relative expression of total *MYOD1* and endogenous *MYOD1* (c). Differentiated myogenic cells derived from mesodermal cells with or without MYOG for 60 days were immunostained with anti-MYOSIN HEAVY CHAIN (MYHC, green) antibody. Nuclei were stained with 4′6-diamidino-2-phenylindole (DAPI, blue). Scale bar, 100 *μ*m (d). Relative expressions of *MYOD1* and *MRF4* transcripts in wild-type or *MYOG-*mutated myogenic cells derived from mesodermal cells (e). All error bars indicate ±SEM (*n* = 3). *P* values are determined by a *t*-test from a two-tailed distribution. ^∗^*P* < 0.05, ^∗∗^*P* < 0.01.

Transient overexpression of *MYOD1* might have overcome the effect of MYOG deficiency because artificially high MYOD1 may compensate the inactivation of the *MYOG* gene in human myogenic cells. To avoid excessive MYOD1 levels, myogenic cells were induced from mesodermal precursors derived from hiPS cell clone number 28, without administration of Dox as shown in Figure 4(a).

The percentage of mesodermal induction marked by DLL1 [22] was shown by FACS analyses and was similar irrespective of *MYOG* mutation (Figure 4(b)). In myogenic cells derived from mesodermal precursors, total *MYOD1* transcripts did not accumulate, in contrast to Dox-treated hiPS cells, including lower level of endogenous *MYOD1* expression (Figure 4(c)). Under these conditions, MYHC-positive differentiated myofibers derived from both MYOG-positive and MYOG-negative hiPS cells were identified to a similar extent (Figure 4(d)). To analyze myogenic differentiation potential from mesodermal cells, transcripts of myogenic regulatory factors were monitored in these cells. The level of *MYOG* transcript was attenuated; however, *MYOD1* or *MRF4* transcripts were not much changed in wild-type and *MYOG*-mutated myogenic cells, as upregulated in *MYOG*-mutated cells during periods of cell culture (Figure 4(e)).

## 4. Discussion

Here, we report the generation of *MYOGENIN*-deficient hiPS cells and the impact on human myogenic differentiation using CRISPR/Cas9 technology. This bacterial system has emerged as an effective tool for gene targeting through nonhomologous end joining (NHEJ); however, it has been reported to be inefficient for precise editing of genomic sequences. In this study, we selected the sequence of *MYOG* exon1-targeted sgRNA with the Cas9 complex as a unique in genomic sequence, which targeted *MYOG* by the T7EI assay not with high efficiency; however, the result of genomic editing in hiPS cells showed high efficiency for knocking out the *MYOG* gene, including in heterozygotes with an efficiency of over 80%. This was not changed with additional azidothymidine, which has been reported to increase the efficiency for NHEJ [25] (not shown).

While knockout mice of *Myog* exhibit a lethal deficiency of differentiated skeletal myofibers, there are nevertheless residual myofibers in *Myog* mutants [12, 13]. The possible differences between in vivo and in vitro situation of *Myog* mutants could be explained by the selection of a particular route to muscle cell differentiation from Myog-independent lineage in vitro, potentially controlled by MyoD1 and Mrf4 because Mrf4 can drive early myogenic differentiation in the myotome when Myog protein is not initially accumulated [10, 14]. Alternatively, there may be a threshold level of total myogenic regulatory factors required in myoblasts to trigger the terminal differentiation program. We have not observed any deficiencies of myogenic differentiation with *MYOG*-mutated cells under two different conditions, either with overexpression of MYOD1 or through medium conditions that promote mesodermal cell progression towards myogenesis. Mutated hiPS cells without not only *MYOG* but also other myogenic factors, *MYF5*, *MYOD1*, and *MRF4*, would be necessary for further analyses to identify the relationships of human myogenic regulatory factors because we observed the upregulation of other myogenic factors in *MYOG*-mutated cells which might compensate MYOG functions in vitro, and triple knockout of *Myog*, *Mrf4*, and *Myod1* or *Myf5*, *Myod1*, and *Mrf4* exhibited impaired ability to terminally differentiate into myofibers, not double knockout of *Myod1* and *Mrf4* [8, 10]. Moreover, there is also other possibility that Myog via skeletal muscle affects systemic factors in vivo [13] and that this feeds back on myofiber formation.

Taken together, these results demonstrate that *MYOG*-mutated human iPS cells have the capacity for myogenic differentiation and can form terminally differentiated myofibers, under differentiation conditions, in contrast to results on developing mouse *Myog* mutants.

## Conflicts of Interest

No competing financial interests exist.

## Figures and Tables

**Table 1:** Candidates and oligos for *MYOGENIN* exon1 target positions for CRISPR/Cas9.

Position		Target sequence	Sequence information		Number of target sites		
Start–end	+/−	20 bp + **PAM**	GC (%)	Tm (°C)	20 bp	12 bp	8 bp
143–165	−	**cct**gcctgtccacctccagggct	70	84	1	61	5171
147–169	−	**cct**gtccacctccagggcttcga	65	80	1	38	92,481
152–174	−	**cca**cctccagggcttcgaaccac	65	79	1	89	10,513
155–177	−	**cct**ccagggcttcgaaccaccag	65	79	1	8	11,331
156–178	+	ctccagggcttcgaaccacc**agg**	65	79	1	1	4654
158–180	−	**cca**gggcttcgaaccaccaggct	65	81	1	7	21,861
166–188	+	tcgaaccaccaggctacgag**cgg**	60	77	1	10	12,349
**170**–**192**	**+**	accaccaggctacgagcgga**cgg**	65	82	1	1	3812
171–193	−	**cca**ccaggctacgagcggacgga	70	82	1	8	1537
174–196	−	**cca**ggctacgagcggacggagct	70	83	1	3	479
191–213	+	**gga**gctcaccctgagccccgagg	75	84	1	43	382

pX458-*hMYOG*+189_F primer: CACCaccaccaggctacgagcgga. pX458-*hMYOG*+189_R primer: AAACtccgctcgtagcctggtggt.

**Table 2:** Primer sequences for T7 endonuclease assay and quantitative RT-PCR.

Genes	Sequences	Amplicon size
Primer for T7 EI assay		
*MYOG* gDNA_F	5′-GGCCGCCCAGCTAGGAGTAATTGA-3′	786
*MYOG* exon1_R	5′-CGCTCGATGTACTGGATGGCACTG-3′	
Primer for RT-qPCR		
*RPL13A*_F	5′-CCCTGGAGGAGAAGAGGAAA-3′	91
*RPL13A*_R	5′-ACGTTCTTCTCGGCCTGTTT-3′	
*MYOG*_F	5′-GCTCAGCTCCCTCAACCA-3′	94
*MYOG*_R	5′-GCTGTGAGAGCTGCATTCG-3′	
*MYOD1*_F	5′-GCACTACAGCGGCGACTCC-3′	118
*MYOD1*_R	5′-GTAGGCGCCTTCGTAGCAG-3′	
Endo-*MYOD1*_F	5′-CACTCCGGTCCCAAATGTAG-3′	180
Endo-*MYOD1*_R	5′-TTCCCTGTAGCACCACACAC-3′	
*MRF4*_F	5′-GGCCAAGTGTTTCCGATCAT-3′	89
*MRF4*_R	5′-AAGGCTACTCGAGGCTGACG-3′	
